# Supplementary material for: Parametric CAD modeling for open source scientific hardware: Comparing OpenSCAD and FreeCAD Python scripts
Source: PLoS One. 2019 Dec 5;14(12):e0225795. doi: 10.1371/journal.pone.0225795 (PMC6894851; doi:10.1371/journal.pone.0225795)
Supplement: Attachment — Submitted filename: response_reviewers.pdf [file pone.0225795.s001.pdf]

Dear reviewers,

We thank you for your time and your comments on the manuscript. We have edited the manuscript to address your concerns.

We apologize about the bibliography of the first version of the manuscript. We used the provided Latex template and believed that it would automatically format the bibliography in compliance with the Journal instructions. However, it seems that it missed some required fields for references that are not journal articles, such as doi for books and technical reports or the access time for webpages. We should have double-checked the result with the required PLOS format before sending the manuscript. We have corrected the bibliography in this revised version.

We have addressed each of your comments in a point-by-point fashion. The comments were very helpful.

Sincerely yours.

Felipe Machado
felipe.machado@urjc.es
Area of Electronics Technology
Universidad Rey Juan Carlos, Móstoles, Spain

Reviewer #1: *The manuscript mainly presents an analysis study of OpenSCAD and compare it with FreeCAD Python scripts. The authors also created a parametric open source hardware design to compare these tools.*

The manuscript is of interest to the PlosOne readership. The introduction provides sufficient background and include all relevant references. The research design is appropriate. The methods adequately described. The results clearly presented. The scientific style of presentation is high enough.

However, the introduction is too long, I would recommend authors to summaries the motivation.

> We have attempted to reduce the introduction length, but since other reviewer believed that the introduction was adequate, we were afraid to remove important background information. Thus, we have barely been able to reduce it.

(Line 73) The authors mentioned some other FOSS CAD tools available to create solid models using a programming language, such as FreeCAD, PythonOCC, CadQuery and BRL-CAD. Among these CAD tools, Among these CAD tools, we have found FreeCAD to be the most active, having around a major release each year,..etc. Could you please compare the interesting features of OpenSCAD and FreeCAD with FreeCAD, PythonOCC, CadQuery and BRL-CAD.

> The reviewer's comment is relevant. We have created a new section with an extended explanation of the features of these FOSS CAD tools. In this section we have explained why we have chosen FreeCAD and also have included the FreeCAD CadQuery workbench in the subsequent analysis. This FreeCAD workbench facilitates the use of Python scripts in FreeCAD, and thus, supports the use of FreeCAD. We believe that with this addition the manuscript is more comprehensive. Besides, we have included the CadQuery models in the software repository.

*(line 262) *.scad*

> Corrected.

The main references are WWW, we recommend the authors to cite research articles if applicable. The readership usually couldn't reach web sites for some cases and that happens for many reasons for example changing/removing the web address when a company acquired by another compony.

The flowing references are a web address; 2, 3, 4, 7, 12, 14, 21,22,30, 34, 3,36,37,38,39,40, 43, 44, 45, 46, 47, 48, 49, 50, 51, 52, 53, 54, 56, 57, and 58.

> The WWW are mainly from computer programs, associations and projects statistics that we have not been able to find research articles with that information. However, all the WWW references have been archived and should be available at any time with the information at the time of archive.

Reference [5] missing the doi.

> Corrected. In the revised manuscript, this reference is [4].

Reference [9] missing the doi.

> Corrected. In the revised manuscript, this reference is [8].

Reference [13] missing the doi.

> We could not find the doi, but we added where it is available. In the revised manuscript, this reference is [12].

Reference [15] is a book but missing the doi.

> We could not find the doi, but the ISBN (9780133373905) could be added if necessary. In the revised manuscript, this reference is [14].

Reference [16] please check the order of the authors; Luis Felipe Rosado Murillo then Matti Veikko Pietari Kauttu.

> Corrected. In the revised manuscript, this reference is [15].

Reference [32] missing doi. doi.org/10.1007/978-3-662-44468-9_53.

Corrected.

Reference [42] missing doi. 10.1201/9781315119601.

Corrected. In the revised manuscript, this reference is [53].

Reviewer #2: After reviewing the manuscript entitled “Parametric CAD modeling for open source scientific hardware: comparing OpenSCAD and FreeCAD Python scripts” (PONE-D-19-22759).

Here are my reviewing points:

The manuscript comparing OpenSCAD and FreeCAD. The work did provide some insight that can potentially guide the developers to improve the CAD modeling using open source scientific hardware.

1. The comparison was comprehensive and the authors provided sufficient resources.

2. The authors provided sufficient and good literature background.

3. The featured of both OpenSCAD and FreeCAD were analyzed fairly.

4. The major drawback is that this manuscript fits to be a case study rather than a research article. However, in both cases it is still valuable and interesting.

5. typos line (73) (The) should be (There).

Corrected.

6. In table (7), the authors mentioned that it is divided into (three) topics, but it seems that there are (four) topics discussed in the table as mentioned in line (238), which are (the geometric modeling kernel, the usability, the programming language characteristics and the tool features), so its need to be corrected to (four) topics in the title of the table.

Corrected.

7. The authors have to follow one format for the references, and I would suggest them to mentioned the access time for websites.

The format of the references have been corrected. We have added the archived link with its archive date in order to have a permanent link at the time of archive.

Reviewer #3: I know several groups working on parametric CAD modeling for open source scientific hardware and they demonstrated progress however there is no single comprehensive study compares such type of CADs (in my knowledge).

The authors present an analysis study of prevalent modeling CADs (OpenSCAD and FreeCAD). They created a parametric open source hardware design to compare OpenSCAD and FreeCAD . The authers also describe methodology of comparison and they provided necessary scripts and supplementary documents.

The main advantage of this paper is the systematic analysis. It feels that the authors predicted readership's questions, so they put consequent answers. The paper is of interest for many PlosOne's auditory. The done works of other groups and researchers are accurately acknowledged, I would recommend to publish this manuscript after minor corrections.

My main concern is about the motivation for the authors to publish their study in a journal while they have already deposited scripts elsewhere (github). I would recommend authors to explain that in the introduction.

> We have deposited the scripts in github to be in compliance with PLOS ONE software sharing policy: *"We expect that all researchers submitting to PLOS submissions in which software is the central part of the manuscript will make all relevant software available without restrictions upon publication of the work".*
>
> In the third section (Open hardware models created as test-bench), in line 172 of the revised marked-up copy, we have included an explanation about the availability of the associated scripted models.

The bibliography needs some attention according to the journal instructions.

> Corrected.
